# Database of ligand-induced domain movements in enzymes

**DOI:** 10.1186/1472-6807-9-13

**Published:** 2009-03-06

**Authors:** Guoying Qi, Steven Hayward

**Affiliations:** 1 School of Computing Sciences, University of East Anglia, Norwich, NR4 7TJ, UK; 2 School of Biological Sciences, University of East Anglia, Norwich, NR4 7TJ, UK

## Abstract

**Background:**

Conformational change induced by the binding of a substrate or coenzyme is a poorly understood stage in the process of enzyme catalysed reactions. For enzymes that exhibit a domain movement, the conformational change can be clearly characterized and therefore the opportunity exists to gain an understanding of the mechanisms involved. The development of the non-redundant database of protein domain movements contains examples of ligand-induced domain movements in enzymes, but this valuable data has remained unexploited.

**Description:**

The domain movements in the non-redundant database of protein domain movements are those found by applying the DynDom program to pairs of crystallographic structures contained in Protein Data Bank files. For each pair of structures cross-checking ligands in their Protein Data Bank files with the KEGG-LIGAND database and using methods that search for ligands that contact the enzyme in one conformation but not the other, the non-redundant database of protein domain movements was refined down to a set of 203 enzymes where a domain movement is apparently triggered by the binding of a functional ligand. For these cases, ligand binding information, including hydrogen bonds and salt-bridges between the ligand and specific residues on the enzyme is presented in the context of dynamical information such as the regions that form the dynamic domains, the hinge bending residues, and the hinge axes.

**Conclusion:**

The presentation at a single website of data on interactions between a ligand and specific residues on the enzyme alongside data on the movement that these interactions induce, should lead to new insights into the mechanisms of these enzymes in particular, and help in trying to understand the general process of ligand-induced domain closure in enzymes. The website can be found at:

## Background

Enzymes are flexible molecules that change conformation upon ligand binding [1,2]. However, there is considerable variation in extent of that conformational change. A database study has shown that movements in enzymes upon substrate binding are generally small [3]. However, another recent study has shown that the extent of movement may depend on the actual reaction mechanism [4]. It is the obvious complexity and variability of conformational change that enzymes exhibit upon ligand binding that makes their study so difficult. In order to help overcome this, we report here on a database specifically devoted to enzymes with a domain movement upon ligand binding. Why do we concentrate on domain movements rather than other kinds of conformational change? The main advantage is that domain movements can be well characterized. That means the domains themselves can be defined, their relative movements can be described in terms of interdomain screw axes (hinge axes), and the hinge-bending regions can also be identified. This ability to characterise domain movements together with the fact that they are generally quite large (large rmsd between the two structures) also means it is possible to decouple them from uninteresting conformational differences that may be due to noise or reasons unrelated to the binding event.

In enzymes with a domain movement the standard view is that the ligand binds to the open conformation and subsequently causes it to adopt a closed conformation where the ligand is surrounded by the enzyme in a highly specific environment. There are a number of different models of the kinetics of ligand binding and protein conformational change in domain proteins. One model, applicable beyond just domain proteins, is the "pre-existing equilibrium model"[5]. In this model equilibrium fluctuations of the protein in the ligand-free state allow it to reach conformations close to those of the ligand-bound state and it is to these that the ligand preferentially binds (this process is also referred to as "conformational selection"). In the "diffusion-collision model" [6], rotational diffusion of the domains in the ligand-free protein is unaffected when the ligand binds to one domain (presumably either) until the other domain comes close enough for it to "glue" the domains together in the closed conformation. An induced-fit model, the "sequential model" [7], has the ligand bind first to a dedicated domain, the "binding domain", and the process of closure is driven (downhill in free energy rather than diffusion on a flat surface) through specific interactions between residues on the "closing domain" and the ligand. A more general "model", is one for which the process of domain closure is regarded as being akin to protein folding [7-9]. It has been suggested that sometimes the ligand can mimic a segment of the protein backbone [7], and when it binds it triggers a final round of folding in which the mimic forms secondary structure like interactions with the real protein backbone. Being rather non-specific suggests that the other models could be accommodated within a more general protein folding model.

In this work a domain movement is defined by the DynDom program. The DynDom program takes two atomic structures and analyses the conformational difference between them in terms of a domain movement. It automatically determines domains, hinge axes, and hinge-bending residues. It does this based on movement, not on structure, and is soundly based in rigid-body kinematics. At its heart is the generation of short main-chain segments by use of a sliding window and the calculation of rotation vectors associated with the rotation of these segments between the two structures. By treating the components of these rotation vectors as coordinates in a "rotation space", segments that rotate together, perhaps comprising a rigid domain within the protein will have rotation points co-located. Effectively this means that domains can be identified as clusters of rotation points. The clusters are identified using the k-means clustering method and are modelled as 3-dimensional normal distributions. This allows one to define an "ellipsoid of significance" for each cluster. Rotation points that have the dual property of lying outside the ellipsoids and in moving along the protein chain are from segments that connect the domains, are assigned "bending" rotation points. The residues associated with the bending rotation points are assigned as bending residues. Further details can be found in the DynDom1.50 paper [10]. An exhaustive application of the DynDom1.50 program to crystal structures in the in the Protein Data Bank (PDB) has resulted in a non-redundant database of protein domain movements where 2035 domain movements are distributed amongst 1578 families [11]. Although there are many causes of the conformational changes seen in this data, in this study we have focussed on those cases where the domain movement is induced by the binding of a functional ligand to an enzyme.

The database described here will be of particular use in understanding how the binding of a ligand can induce conformational change. Its key characteristic is the presentation of data related to the binding of the ligand in the context of dynamical features such as the dynamic domains, hinge axes, and the hinge-bending residues. It is the latter that are of particular interest as it is these that collectively control the domain movement [12] and in several cases, have been implicated in being involved in inducing domain closure [7]. Not only will it be of use in understanding ligand induced domain closure in enzymes it will be of use for the development of methods for the prediction of protein flexibility [13-15].

## Construction and content

### Dataset Preparation

Here the methods used to extract domain movements caused by the binding of a functional ligand to an enzyme are described. This involved the selection of enzymes from the non-redundant database of protein domain movements, the selection of those enzymes where a ligand is present in at least one of the structures, the verification of the ligand as a functional ligand, and the final selection of those cases where the ligand could have triggered the conformational change upon binding.

The current DynDom database [11,16] of protein domain motions provides a comprehensive and non-redundant dataset of protein domain movements based on the DynDom (version 1.50) methodology [10,17,18]. Each movement is defined by a pair of homologous protein chains in different conformations solved by X-ray crystallography. The database used here comprised 2035 domain movements from 1578 protein families derived from the March 2007 release of the PDB. Protein regions were divided into domains and bending regions. In order to simplify the analysis, proteins with three or more domains and those with more than ten bending regions were excluded.

#### Domain Movements in Enzymes

Each PDB file was scanned for EC numbers and protein chains were associated with one or more EC numbers. A domain movement was assigned to an enzyme if either or both of its two protein chains had been associated with at least one EC number. The domain movements not associated with any EC numbers or associated with incomplete EC numbers were excluded from the analysis. Out of the initial 2035 pairs, this procedure resulted in 764 pairs being assigned to an enzyme.

#### Domain Movements with Ligands

For each protein chain, there may be one or more ligands in its PDB file. Some of these ligands have the same chain ID as the protein chain. These ligands were associated directly to the protein chain. However, some ligands in the PDB file do not have a chain ID. In this case those ligands were provisionally associated to all the protein chains in the PDB file. For each protein chain this process resulted in a list of "PDB ligands". All domain movements were excluded from the dataset if both protein chains had an empty list. This list (one for each chain) is termed the "PDB-ligand list". This procedure reduced the dataset down to 693 pairs.

#### Functional Ligands

The following procedure was carried out in order to ensure that the PDB ligand was a functional ligand for the enzyme, possibly able to induce a functional domain movement. In order to determine whether the PDB ligands associated with each protein chain were functional ligands, the Kyoto Encyclopaedia of Genes and Genomes (KEGG) LIGAND database for enzymes [19] was used. In the KEGG LIGAND database, a list of compounds (substrates, products, cofactors, coenzymes and inhibitors) is given for each enzyme, as identified by its EC number. For each protein chain a list of all the compounds was compiled for its assigned EC number(s). This list (one for each chain) was termed the "KEGG-ligand list". For each protein chain, its PDB-ligand list was matched to its KEGG-ligand list by cross-checking for similar chemical formulae. If the difference in the number of heavy atoms between the two formulae were less than or equal to two, a match was assigned, meaning that the PDB ligand was considered to be a functional ligand for this protein chain. A mismatch of two heavy atoms was thought to be sufficiently strict not to result in too many false positives being included, but sufficiently lax so as not to result in too many false negatives being rejected. The resulting list of functional ligands for each protein chain is termed the "functional-ligand list". Domain movements where both protein chains had an empty functional-ligand list were removed. Out of the 693 domain movements, only 360 survived this procedure. It was at this stage that domain movements with more than two domains were removed as were those remaining with more than ten bending regions. This left 312 domain movements.

#### The Contact-ligand Set

For each protein chain all ligands in the functional-ligand list not in contact with the protein in either conformation were removed. Subsequently those protein pairs without any ligands were removed. The remaining pairs formed the "contact-ligand set" and the remaining ligands the "contact ligands". The contact-ligand set comprises all those protein pairs with at least one ligand contacting the protein in either conformation. Here and below "in contact" means that the ligand has a heavy atom within 4 Å of a heavy atom of the protein. Of the 312 domain movements from the previous stage, a further 14 were removed by this process leaving 298.

#### Spanning Ligands and Non-spanning Ligands

The classic view for a ligand-induced domain closure in an enzyme is one where the ligand binds in the interdomain cleft and is surrounded by the protein. If a ligand is in contact with one domain as well as in contact with the other domain, or bending regions, or both, then the ligand will be termed a "spanning ligand". All other contact ligands are "non-spanning ligands".

#### Trigger-ligand, Spanning Trigger-ligand and Non-spanning Trigger-ligand Sets

**Figure 1:**
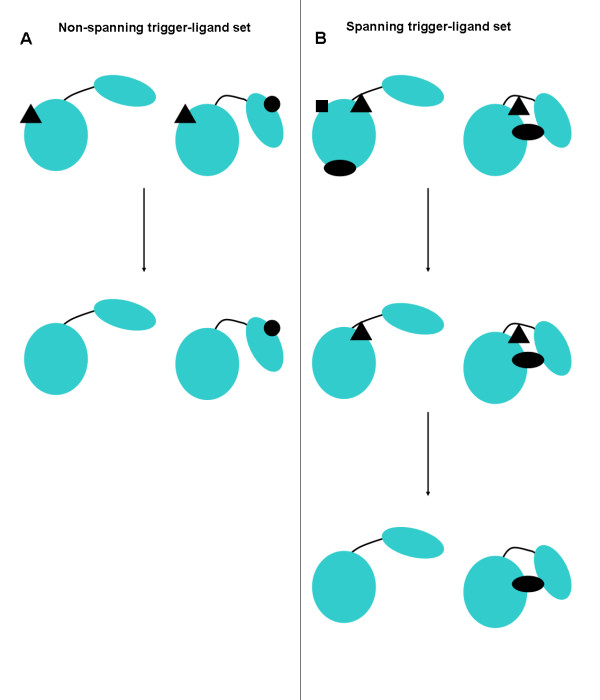
**Procedure used to determine the trigger-ligand set**. Two-domain proteins are depicted with a bending region, indicated as a curved line, linking two domains drawn as large ellipses. Different contact ligands (which have also been confirmed using KEGG to be functional ligands) are indicated in black as filled circles, triangles, squares, and ellipses. The contact ligand set is divided into two groups: those without a spanning ligand and those with a spanning ligand. A spanning ligand is one that is in contact with one domain as well as in contact with the other domain, or bending regions, or both. **(A) The non-spanning trigger ligand set: **Identical ligands in both conformations are removed (i.e. the triangle ligand) to leave the circle ligand as the non-spanning trigger-ligand. This pair of conformations is put in the non-spanning trigger-ligand set. **(B) The spanning trigger ligand set: **First non-spanning ligands are removed from both conformations (i.e. the ellipse and the square ligands on the conformation on the left), to leave the triangle ligand and the ellipse ligand both of which are spanning ligands. Then, identical ligands in both conformations are removed to leave the ellipse ligand as the spanning trigger-ligand. This pair is put in the spanning trigger-ligand set. Combined, the non-spanning trigger-ligand set (53 examples) and the spanning trigger-ligand set (150 examples) form the trigger-ligand set (203 examples).

The basic concept of a "trigger ligand" is that it should be a contact ligand that is present in one conformation but not the other, i.e. it has caused the conformational change upon binding to the enzyme. However, if a ligand is in both conformations, but is spanning in one conformation but not the other, then it is also considered to be a trigger ligand. The procedure used, therefore, is one that gives priority to spanning ligands over non-spanning ligands. This is reasonable in the light of what is known about ligand-induced domain closure, i.e. the ligand is usually enclosed by an enzyme with a domain movement. The procedure to determine the "trigger-ligand set", illustrated in Figure 1, first checks for spanning ligands in either conformation dividing into two groups: those with spanning ligands and those without. For the group without, identical ligands (tested by ligand name matching between PDB files) in both conformations were removed, and those pairs remaining that had one conformation with a contact ligand (the "non-spanning trigger ligands"), and the other without, were put in the "trigger-ligand set". For the group with spanning ligands, all non-spanning ligands were removed. Then identical ligands in both conformations were also removed. The remaining pairs that had one conformation without a ligand and the other with a spanning ligand (the "spanning trigger-ligand") were added to the trigger-ligand set. Thus the trigger-ligand set is the union of two non-overlapping sets, the "non-spanning trigger-ligand set" and the "spanning trigger-ligand set". This final procedure removed 95 pairs leaving 203 pairs in the trigger-ligand set, 53 from the non-spanning trigger-ligand set and 150 from the spanning trigger-ligand set.

The following section describes analyses performed on the 203 pairs in the trigger-ligand set where the domain movement is apparently triggered by the binding of a functional ligand.

### Dataset Analysis

#### Contacts between ligand and Extended Bending Regions

In the previous study [7], it was found that ligands often contact interdomain bending regions or their immediate neighbours. "Extended bending regions" were defined as bending regions plus three residues either side.

#### Hydrogen Bonds and Salt-bridges between Ligand and Enzyme

In order to determine residues making hydrogen bonds and salt bridges with the ligand, the program LIGPLOT was used [20]. LIGPLOT is a program which can automatically generate schematic diagrams of protein-ligand interactions given a PDB file based on the hydrogen bonds, salt-bridges and hydrophobic contacts calculated by another program HBPLUS [21]. LIGPLOT was used as a harness for running HBPLUS. LIGPLOT was run on each ligand bound conformation of each pair in the trigger-ligand set to produce a list of hydrogen bonds and salt-bridges between the trigger ligand and the protein.

#### Radius of Gyration

Given the analogy of ligand-induced domain closure with protein folding the radius of gyration of the ligand bound and ligand unbound conformations was calculated. The radius of gyration was calculated using backbone atoms with any insertions indicated by a pairwise sequence alignment excised from the structures.

### Integration of Data into Existing Database and Display at Website

#### Database Design

**Figure 2:**
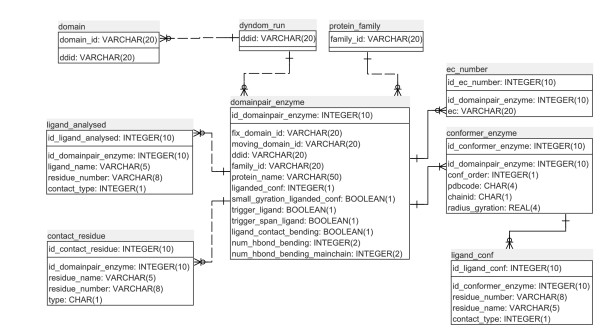
**Database Entity Relationship Diagram**. The top row of each table is the primary key, and all of the relationships are one-to-many. The three tables at the top of the figure, "protein_family", "dyndom_run", and "domain" existed prior to this analysis. Listed in each table are the attribute names together with data type. The names are self-explanatory in most cases. In the "contact-residue" table, the attribute "type" categorises the interaction type (e.g. a hydrogen bond or just a contact), its location (e.g. in an extended bending region), and whether the hydrogen bond is with the side chain or main chain. In the "ligand_analysed" table and the "ligand_conf" table the "contact_type" attribute indicates whether the ligand is spanning or non-spanning. See main text for further explanation.

The results of this analysis occupy six tables in the DynDom database of protein domain motions. Figure 2 shows the database schema. The three tables at the top of the figure, "protein_family", "dyndom_run", and "domain" existed prior to this analysis and are part of a larger set of tables in the relational database [11]. The main table is the "domainpair_enzyme" which contains general data on ligand contacts and conformation. It links to the protein_family, dyndom_run, and domain tables from which further details of the protein family members and data of the domain movement itself can be accessed. It also links directly to four other tables which store details of the ligands (ligand_analysed), contacting and hydrogen-bonding residues (contact_residue), the EC number of the enzyme (ec_number) and data on each conformation of the pair that represent the domain movement (conformer_enzyme). A link from the latter table connects to the "ligand_conf" table which contains data on all the ligands for that structure as found in its PDB file.

#### Presentation at Website

The web-interface to the database is implemented using JAVA Server Pages (JSP) and servlets. The database software itself is PostgreSQL. The front page lists the enzymes giving their names, EC numbers, PDB accession codes and chain identifiers for each protein pair. In addition the front page indicates whether the ligand is a spanning ligand or not, and whether the ligand has caused compaction of the proteins upon binding. For each pair there is a link to its main page. At the top of the main page the structure of the ligand bound state is displayed using the molecular graphics applet, Jmol . The protein structure is coloured according to domain (blue or red) and interdomain bending regions (green). The trigger-ligand is displayed in spacefilling model and the contacting residues are indicated in ball and stick model. Below this there are two Jmol windows displaying the domain movement in relation to the ligand. These two displays correspond to the two alternative scenarios of the sequential model [7]: one where the ligand binds first to domain 1 before closure is induced, the other where it binds first to domain 2 before inducing closure. In the former case domain 1 is the binding domain and 2 the closing domain, and in the latter it's vice-versa. The procedure used to construct these models is described in [7]. They show the ligand bound to the binding domain, both of which are held fixed in space, and the closing domain as the moving domain closing upon them.

The following section shows a sequence alignment of the two chains coloured according to domain and bending region. The sequences between the two chains need not be identical but according to the construction of the non-redundant database they will have a 90% or greater sequence identity with the representative of their family [11].

**Figure 3:**
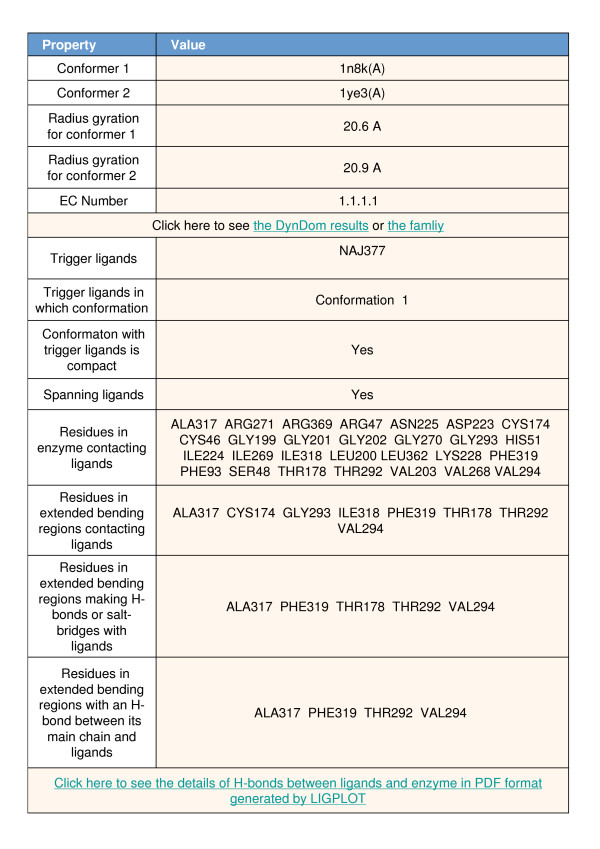
**Main table of webpage for liver alcoholdehydrogenase**. See main text for further explanation.

Next is the main table shown in Figure 3 for liver alcohol dehydrogenase (LADH). It gives details on the ligand-protein interactions and provides links to other pages on the website. At the top of the table are details on the type of ligand (whether spanning or non-spanning) the radii of gyration for the ligand unbound and ligand bound conformers, the ligand name as given in the PDB file, and links to "the family" page and the "DynDom results" page for that pair. The DynDom results page gives details on the DynDom run, on the domain decomposition and the bending residues, on the movement (e.g. the rotation angle and translation along the hinge axis), and through another link details on dihedral angle changes that occur at the bending residues. The family page gives all other members of that family and presents details of a conformational clustering process and the set of representative movements of the family, one of which is the ligand-induced movement concerned. Thus although the movement considered in this study is between two structures only and may not therefore be representative of all the important modes of motion for the enzyme, other modes of motion may be found on its family pages. Further details can be found in Qi et al. [11].

Other sections of the main table list the residues that contact the ligand, residues in extended bending regions that make a hydrogen bond or salt-bridge with the ligand and residues in extended bending regions with a hydrogen bond between their main chain and the ligand. Using the sequence alignment one can identify the equivalent residues in the ligand unbound chain. Finally there is a link to a LIGPLOT, where a schematic diagram shows all the interactions between the ligand and individual residues.

## Utility and discussion

To our knowledge this is the only web-accessible database for enzymes that provides ligand-binding information in a dynamical context. Its primary aim is to help researchers in understanding ligand-induced conformational change in enzymes. The dataset accumulation was necessarily different from previous studies [3,4] as it originates from structural pairs displaying a domain movement. The study by Gutteridge and Thornton [3] started from a set of enzymes annotated in the catalytic site atlas (CSA) [22] which they refined down to a set of structures classified as: apo, some substrates bound, all substrates bound, transition state bound, all products bound, some products bound or unclassifiable. This was filtered further using a resolution cut-off of 2.5 Å and non-redundant filtering using CATH number [23]. Koike et al. [3,4] selected just monomeric proteins with the ligand bound and unbound structures having at least 95% sequence identity. They also checked that the ligand was in the vicinity of active site residues by cross referencing with Uniprot [24]. Both these two previous database studies resulted in about 60 pairs. It appears that their datasets have not been made available through a website.

As mentioned in the Background section we have concentrated on domain movement as unlike other conformational changes in proteins, they can be characterised through the methods of rigid-body kinematics, e.g. the relative movement of the domains can be described by a screw movement according to Chasles' theorem [25]. An understanding of the movement, combined with a set of interactions between the ligand and the enzyme should give insight into how these interactions can cause the observed conformational change. Of particular interest are interdomain bending regions. It is known that these control the domain movement as the hinge axis is often seen to pass close to them, much like the hinge axis of a door passes through the hinges that attach it to a wall [12]. In five enzymes having a domain movement it was also found that the ligand interacted (formed hydrogen bonds in two cases, formed a cation-pi interaction in one case, and more general electrostatic interactions in two cases) with residues on bending regions or their near neighbours [7]. For this reason we give information at the website on residues in the extended bending regions that have a specific interaction (a hydrogen bond or salt-bridge) with the ligand. This information should be of help in understanding why in general, and why in specific cases, ligands interact with hinge-bending regions.

### Case Study LADH

Let us consider LADH as an example of how the data at the website might lead to an understanding of the relationship between the ligand-enzyme interactions and the domain movement. LADH is an enzyme that catalyses the oxidation of alcohol to aldehyde and is a homodimer, with each protomer comprising a coenzyme binding domain and a catalytic domain. The binding of NAD+ in the interdomain cleft induces domain closure preparing the enzyme for the binding of the alcohol substrate [26-28]. The LADH page is given at:  but its main table is also shown in Figure 3. The conformational pair are the A chains from PDB files 1N8K and 1YE3. 1N8K(A) has four ligands (4s)-2-methyl-2,4-pentanediol (MDP in PDB 3 letter code), nicotinamide-adenine-dinucleotide, NAD, (NAJ in PDB code), pyrazole (PZO) and zinc (ZN). 1YE3(A) chain has MDP and ZN as its ligands. The procedure used to identify the ligand that triggers the domain movement has correctly selected NAD as the trigger ligand, which, as indicated on the main page is in conformer 1, identified as 1N8K(A). Thus the ligand unbound conformation is 1YE3(A) and the ligand bound conformation is 1N8K(A). As one can see from other rows in the table the NAD ligand is a spanning ligand and it has caused compaction of the protein upon binding.

Following "the family" link on row 6 of the table one finds that there are a total of 73 structures in the non-redundant database belonging to the same LADH family. Through conformational clustering 1N8K(A) has been selected as the representative of one cluster (the closed structure) comprising 62 structures, and 1YE3(A) the representative of another cluster (the open structures) comprising 11 structures [11]. Following the "DynDom results" link leads to information on the DynDom run itself, domain definitions and a section with details of the domain movement. In this case the angle of rotation is 8.5° which is accompanied by a -0.3 Å translation along the axis. The motion itself has been classified as a 95.6% closure[18].

Rows 11–14 detail specific residues contacting or making hydrogen bonds or salt-bridges with the NAD ligand. We find an appreciable number of residues in extended bending regions that make a hydrogen bond with the NAD ligand. In particular we find hydrogen bonds between the ligand and the main chain of extended bending residues Ala317, Phe319, Thr292 and Val294. The nicotinamide group of NAD forms hydrogen bonds with the Ala317 and Phe319 which are situated at the terminus of a β-sheet (this can be determined using the Jmol display at the top of the page) that would appear to mimic those found in a true β-sheet [7]. The interaction between Val294 has been found to be central to the switch mechanism operating in LADH by stabilising the loop in conformation that allows the domains to close [29]. All the interactions between the protein and the ligand are visualised schematically in the LIGPLOT link. Thus by focussing in on extended bending regions that interact with the ligand, some key residues involved in the mechanism of domain closure in LADH could be identified.

This example illustrates that putting ligand binding information in a dynamical context can lead to the identification of key residues involved in inducing domain closure.

### General Analysis of Data

Given that interdomain bending regions control the domain movement it is interesting to know in what proportion of cases there is a contact or specific interaction between the trigger-ligand and an extended bending region. It was found that out of the 298 domain pairs in the contact-ligand set, 59% of pairs have ligands contacting extended bending regions. For the 203 pairs in the trigger-ligand set, 66% of ligands contact an extended bending region, but for the spanning trigger-ligand set, the corresponding figure is 84% and for the non-spanning trigger-ligand set it is only 13%. Table 1 gives a breakdown of contacts and hydrogen bonds or salt-bridges between the ligand and residues in extended bending regions amongst the sets. A considerable proportion (104/150, 69%) of the spanning trigger-ligand set, has at least one hydrogen bond/salt bridge between the ligand and a residue in an extended bending region. Of these 104 cases, 52% have a hydrogen bond with the main chain.

**Table 1:** General analysis of interactions between extended bending regions and ligand

Set	Number in set	Number in contact with extended bending region	Number making a hydrogen bond or salt-bridge with extended bending region	Number hydrogen bonding with main chain of extended bending region
Spanning trigger-ligand	150	126 (84%)	104 (69%)	55 (37%)

Non-spanning trigger-ligand	53	7 (13%)	6 (11%)	3 (6%)

Trigger-ligand	203	133 (66%)	110 (54%)	58 (29%)

**Figure 4:**
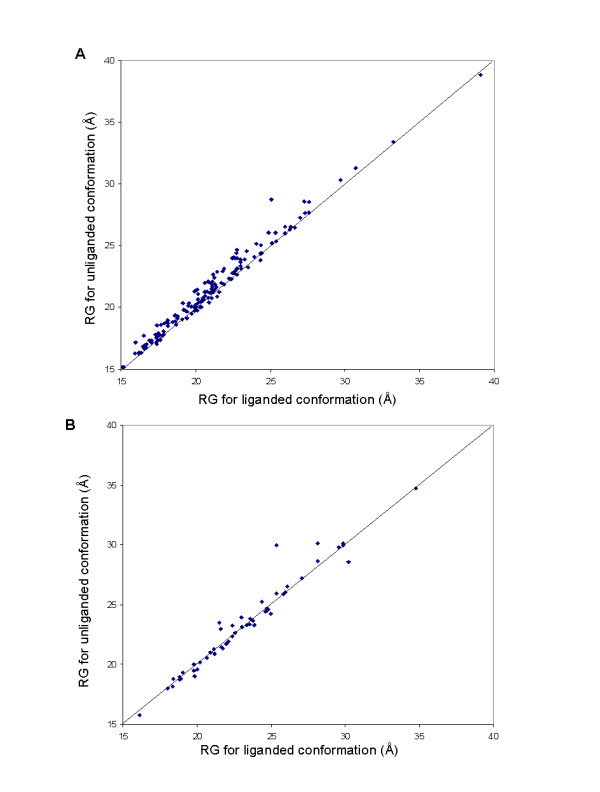
**Radius of gyration plots of ligand unbound conformation against ligand bound conformation**. **(A) **For the spanning trigger-ligand set. **(B) **For the non-spanning trigger-ligand set.

If domain closure is like protein folding then one would expect the ligand bound conformation to be more compact than the ligand unbound. Although, anecdotally, one expects that the ligand bound conformation should be more compact than the ligand unbound, to our knowledge no study has demonstrated this. The radius of gyration was calculated for each pair in the spanning trigger-ligand set and the non-spanning trigger-ligand set. Figure 4A shows the result of this analysis on the former where in 78% of cases (117/150) the binding of the ligand results in a more compact structure. In the latter case, however, shown in Figure 4B, there is no trend for compaction (26 cause compaction, 27 expansion). The outlier in Figure 4B is tyrosyl-tRNA synthetase. Thus the basic concept of compaction of the enzyme upon ligand binding is generally true for the spanning trigger ligand set but not the non-spanning trigger-ligand set.

### Future Developments

For some examples in the non-spanning trigger-ligand set, the trigger ligand is bound to a region remote to the interdomain cleft. Although one can think of complex mechanisms that might explain how binding at these remote sites can initiate domain closure, in some of these cases the trigger ligand may not be the true initiator of domain closure. We therefore welcome comments from expert users on this issue. In the future, based on this expert knowledge, we plan to build a filtered version of the database.

## Conclusion

A new database has been described that presents ligand binding information in the context of dynamical information for enzymes that exhibit a domain movement upon ligand binding. The 203 domain movements in the database were derived using a careful data-filtering procedure applied to the 2035 domain movements that comprise the non-redundant database of protein domain movements. The database will be of particular use to experts interested in a particular enzyme present in the database. Alongside other studies it will also be of use in understanding how ligands induce conformational changes in enzymes in general, and which of the kinetic models of ligand-induced domain closure is most appropriate.

## Authors' contributions

GQ did the programming, data handling and constructed the website. SH and GQ both participated in the development of the methods that created the dataset and wrote the paper. SH had the original idea.

## References

[B1] Hammes GG (2002). Multiple conformational changes in enzyme catalysis. Biochemistry.

[B2] Teague SJ (2003). Implications of protein flexibility for drug discovery. Nat Rev.

[B3] Gutteridge A, Thornton J (2005). Conformational changes observed in enzyme crystal structures upon substrate binding. J Mol Biol.

[B4] Koike R, Amemiya T, Ota M, Kidera A (2008). Protein structural change upon ligand binding correlates with enzymatic reaction mechanism. J Mol Biol.

[B5] Ma BY, Shatsky M, Wolfson HJ, Nussinov R (2002). Multiple diverse ligands binding at a single protein site: A matter of pre-existing populations. Protein Sci.

[B6] Gerstein M, Lesk AM, Chothia C (1994). Structural mechanisms for domain movements in proteins. Biochemistry.

[B7] Hayward S (2004). Identification of specific interactions that drive ligand-induced closure in five enzymes with classic domain movements. J Mol Biol.

[B8] Dobson CM (1990). Protein Conformation – Hinge-Bending and Folding. Nature.

[B9] Kumar S, Ma BY, Tsai CJ, Wolfson H, Nussinov R (1999). Folding funnels and conformational transitions via hinge-bending motions. Cell Biochem Biophys.

[B10] Hayward S, Lee RA (2002). Improvements in the analysis of domain motions in proteins from conformational change: DynDom version 1.50. JMol Graph Model.

[B11] Qi G, Lee RA, Hayward S (2005). A comprehensive and non-redundant database of protein domain movements. Bioinformatics.

[B12] Hayward S (1999). Structural principles governing domain motions in proteins. Proteins.

[B13] Andrusier N, Mashiach E, Nussinov R, Wolfson HJ (2008). Principles of flexible protein-protein docking. Proteins.

[B14] Cavasotto CN, Singh N (2008). Docking and high throughput docking: Successes and the challenge of protein flexibility. Curr Comput-Aid Drug Des.

[B15] Dobbins SE, Lesk VI, Sternberg MJE (2008). Insights into protein flexibility: The relationship between normal modes and conformational change upon protein-protein docking. Proc Natl Acad Sci USA.

[B16] Lee RA, Razaz M, Hayward S (2003). The DynDom database of protein domain motions. Bioinformatics.

[B17] Hayward S, Kitao A, Berendsen HJC (1997). Model free methods to analyze domain motions in proteins from simulation. A comparison of a normal mode analysis and a molecular dynamics simulation of lysozyme. Proteins.

[B18] Hayward S, Berendsen HJC (1998). Systematic analysis of domain motions in proteins from conformational change: New results on citrate synthase and T4 lysozyme. Proteins.

[B19] Goto S, Okuno Y, Hattori M, Nishioka T, Kanehisa M (2002). LIGAND: database of chemical compounds and reactions in biological pathways. Nucl Acids Res.

[B20] Wallace AC, Laskowski RA, Thornton JM (1995). LIGPLOT – A program to generate schematic diagrams of protein ligand interactions. Protein Eng.

[B21] McDonald IK, Thornton JM (1994). Satisfying hydrogen-bonding potential in proteins. J Mol Biol.

[B22] Porter CT, Bartlett GJ, Thornton JM (2004). The Catalytic Site Atlas: a resource of catalytic sites and residues identified in enzymes using structural data. Nucleic Acids Res.

[B23] Pearl FMG, Lee D, Bray JE, Sillitoe I, Todd AE, Harrison AP, Thornton JM, Orengo CA (2000). Assigning genomic sequences to CATH. Nucleic Acids Res.

[B24] Bairoch A, Bougueleret L, Altairac S, Amendolia V, Auchincloss A, Puy GA, Axelsen K, Baratin D, Blatter MC, Boeckmann B (2008). The Universal Protein Resource (UniProt). Nucleic Acids Res.

[B25] Chasles M (1830). Note sur les propriétés générales du système de deux corps semblables entr'eux et placés d'une manière quelconque dans l'espace; et sur le déplacement fini ou infiniment petit d'un corps solide libre. Bulletin des Sciences Mathematiques, Astronomiques, Physiques et Chimiques.

[B26] Eklund H, Samama JP, Wallen L, Branden CI, Akeson A, Jones TA (1981). Structure of a Triclinic Ternary Complex of Horse Liver Alcohol-Dehydrogenase at 2.9 A Resolution. J Mol Biol.

[B27] Eklund H, Samama JP, Jones TA (1984). Crystallographic Investigations of Nicotinamide Adenine-Dinucleotide Binding to Horse Liver Alcohol-Dehydrogenase. Biochemistry.

[B28] Colonna-Cesari F, Perahia D, Karplus M, Eklund H, Branden CI, Tapia O (1986). Interdomain motion in liver alcohol dehydrogenase: Structural and energetic analysis of the hinge bending mode. J Biol Chem.

[B29] Hayward S, Kitao A (2006). Molecular dynamics simulations of NAD+-induced domain closure in horse liver alcohol dehydrogenase. Biophys J.

